# A noise audit of human-labeled benchmarks for machine commonsense reasoning

**DOI:** 10.1038/s41598-024-58937-4

**Published:** 2024-04-14

**Authors:** Mayank Kejriwal, Henrique Santos, Ke Shen, Alice M. Mulvehill, Deborah L. McGuinness

**Affiliations:** 1 Information Sciences Institute, University of Southern California, Marina del Rey, 90292 USA; 2 Rensselaer Polytechnic Institute, Tetherless World Constellation, Troy, New York USA

**Keywords:** Computer science, Human behaviour

## Abstract

With the advent of large language models, evaluating and benchmarking these systems on important AI problems has taken on newfound importance. Such benchmarking typically involves comparing the predictions of a system against human labels (or a single ‘ground-truth’). However, much recent work in psychology has suggested that most tasks involving significant human judgment can have non-trivial degrees of noise. In his book, Kahneman suggests that noise may be a much more significant component of inaccuracy compared to bias, which has been studied more extensively in the AI community. This article proposes a detailed noise audit of human-labeled benchmarks in machine commonsense reasoning, an important current area of AI research. We conduct noise audits under two important experimental conditions: one in a smaller-scale but higher-quality labeling setting, and another in a larger-scale, more realistic online crowdsourced setting. Using Kahneman’s framework of noise, our results consistently show non-trivial amounts of level, pattern, and system noise, even in the higher-quality setting, with comparable results in the crowdsourced setting. We find that noise can significantly influence the performance estimates that we obtain of commonsense reasoning systems, even if the ‘system’ is a human; in some cases, by almost 10 percent. Labeling noise also affects performance estimates of systems like ChatGPT by more than 4 percent. Our results suggest that the default practice in the AI community of assuming and using a ‘single’ ground-truth, even on problems requiring seemingly straightforward human judgment, may warrant empirical and methodological re-visiting.

## Introduction

The advent of large language models and other complex Artificial Intelligence (AI) systems has led to parallel development and release of labeled datasets for evaluating their capabilities^1^. One class of problems that has spurred much interest in such ‘benchmarking’ is machine commonsense reasoning. For practical purposes, commonsense reasoning is minimally defined as a “shared human ability of understanding and navigating everyday situations^2–4^”. An example of a natural language inference problem involving commonsense reasoning, taken from a classic benchmark dataset called the *Winograd Schema Challenge* (WSC)^5^ is “The trophy would not fit in the suitcase because it was too big. What was too big?” Given this prompt, and two options “trophy” and “suitcase,” an AI system would need to pick an option and its overall score on a set of such questions would then be evaluated against a human-labeled ground truth (in this case “trophy”). A unique aspect of WSC that made it challenging before the advent of language models, is that “big” in the question above could be replaced with “small,” leading to the correct answer flipping to the other option. Although commonsense reasoning is a quintessential human ability, it remains controversial to determine whether machines (including the most recent large generative models like GPT-4 and DALL-E) possess it, owing to disagreements about how to measure it comprehensively and reliably^6,7^.

Unfortunately, there has been far less focus on the quality of human-labeled datasets for benchmarking AI performance on commonsense reasoning. In the original publication or release of the benchmark, there is often a presumption of a single ‘ground-truth’ set of labels^8^, despite the fact that human performance itself is known to be non-trivially lower than the maximum theoretically achievable score. For example, human performance on the Social Intelligence Question Answering (SIQA)^9^ benchmark is reported to be at 90 percent or lower, depending on how, and over what partition of the benchmark (i.e., development or test), the human performance was estimated. However, because the ground-truth against which human performance was estimated was *itself* acquired through human labeling, it is controversial to conclude that a typical human test subject is ‘wrong’ on those 10 percent of instances, especially considering the commonsense content of the benchmark (as opposed to specialized content requiring subject-matter expertise, such as in law or medicine). Rather, it may be more appropriate to state that human judgment can exhibit *variability* on those instances.

Variability in human judgment has not gone unnoticed by researchers^10^, both within behavioral psychology and AI. There are two broad causes: *bias* and *noise*. A considerable body of research has been published on the former^11,12^, but there is comparatively little study on the latter. In 2021, Kahneman, Sibony and Sunstein^13^ published a book on noise, where they cite a broad set of examples to conclude that “wherever there is judgement, there is noise.” They define noise as variability in judgments that should be identical and explain how variability exists because judgments are subjective and do not necessarily follow exact rules^14^. The examples therein are derived from an extensive study about how judgement and prediction are made during decision-making in difficult contexts, ranging from criminal-sentencing decisions by judges to settling issues of insurance premiums. In fact, their evidence suggested that noise may be a much greater source of variability in such tasks than bias. A key recommendation of the book is to conduct a *noise audit* in any task where there is a significant dependence on human judgment.

This article proposes a rigorous noise audit of human-labeled machine commonsense reasoning benchmarks. To the best of our knowledge, such an audit has not been conducted before for a set of AI benchmarks, and consequently, quantifying the types and amounts of noise (as defined in Kahneman’s work) remains open. One reason might be that conducting such an audit requires us to obtain multiple judgments (in our case, *labels*) for the same task. Unfortunately, although many benchmarks for evaluating machine commonsense reasoning exist at the time of writing, labeling guidelines and sets of labels independently acquired from multiple individuals (‘annotators’) are not available for the majority of these benchmarks^8,15^. In many cases, the benchmark publication is also vague about whether labels were even acquired by (and aggregated over) more than one annotator. Therefore, rather than do a post-hoc analysis of secondary data, we conduct a detailed noise audit by collecting primary data. We begin by enumerating two specific research questions (RQs) that are investigated in this article:

1. **RQ1:** What are the quantitative outcomes of a *noise audit* conducted on human-labeled benchmark datasets designed to measure machine commonsense reasoning?
2. **RQ2:** How does noise in these benchmarks affect our estimates of systems’ performance (and relative competitiveness) on the commonsense reasoning task?

We recognize that the outcome of RQ1 can be significantly affected by a range of factors, including the manner in which benchmarking is conducted, the dataset is formatted, and so on. While determining the effect of every such potential factor is infeasible, we do aim to consider several reasonable factors in order to ascertain the range and consistency of noise levels. The most important of these is the benchmarking setting itself. We consider two important settings in our study. In the first setting, we perform the labeling exercise in a laboratory setting on a recently released theoretically-grounded commonsense reasoning benchmark (TG-CSR^16^) spanning four thematic areas (called “contexts”), two modalities (formats) and nine commonsense core domains (e.g., time and space). By obtaining results across these contexts, formats and core domains, we can ascertain the stability of our noise audit results and avoid potential dataset bias^12,17,18^. The issue of modality (e.g., whether to provide choices for questions, or to just format them as *true/false*-style questions) is an especially important one for this study, because work by Kahneman et al., as well as other researchers in communities like human–computer interaction, have commented on the impact that such modalities can have on elicitation of human ratings^19,20^. To our knowledge, no other commonsense reasoning benchmark offers the same questions manually formatted using two different modalities (but with the same semantic content). Conducting a comprehensive noise audit on TG-CSR enables us to further evaluate this evidence in the context of machine commonsense reasoning. Furthermore, because questions in TG-CSR span four different thematic areas and are diverse in their coverage of commonsense core domains like time and space, we are also able to check for (and comment on) qualitative consistency in noise audit results across the thematic areas.

In the second setting, we conduct the audit in a more realistic annotation scenario on Amazon Mechanical Turk (AMT) that mirrors documented benchmarking practices in machine commonsense reasoning^9^. AMT is a *crowdsourcing* platform, where ‘micro-tasks’ can be published for crowdsourced workers (called ‘turkers’) to solve for a stated amount of money. We use a set of two thousand sentences released as part of the widely used ComVE benchmark^21^, subsequently described. As is the case for most benchmarks, ComVE was only released with a single ground-truth, without any discussion of noise. Questions in ComVE are short and independent (rather than grouped by thematic area), usually involving a statement in simple English (e.g., “He drinks milk”) and a prompt asking the annotator whether the statement is plausible or implausible. This makes them especially well-suited to be framed as AMT micro-tasks, and allows a turker to label as many, or as few, instances as they want. This allows us to investigate practical questions such as: do turkers who choose to label more prompts exhibit more ‘noise’ in their labels than those who provide fewer labels? And, is noise in an online setting markedly greater (or lower) than in the lab-based setting implemented for TG-CSR? We also comment on the feasibility of designing a similar online noise audit for TG-CSR in the “Limitations and Implications” section, although we leave the actual implementation and analysis of such an audit for future work.

RQ2 investigates the consequences of noise for evaluating the performance of one or more commonsense reasoning systems. We consider the term ‘system’ in the most general sense possible, including not just an AI model like ChatGPT, but also humans. As with RQ1, we consider two situations, one that is more homogeneous and based in a laboratory setting, and another, a more realistic online setting. In the laboratory setting, which applies to TG-CSR, we evaluate how estimates of human performance (often the high point that computational commonsense reasoning systems are benchmarked against^22^) change due to noise. In the online setting, applying to ComVE, we conduct a similar analysis, but using ChatGPT.

Our empirical results show that not only is non-trivial noise prevalent in the labels of both ComVE and TG-CSR, but that the levels of different sub-types of noise are qualitatively consistent between the two, despite significant differences in both setting and format. In investigating RQ2, the experiments show that the noise can lead to different estimates of performance both for humans and for advanced systems like ChatGPT, often by statistically significant margins. We conclude the article by discussing and placing these findings in a broader context.

## Noise audit framework: definitions and notation

In their book “Noise: A Flaw in Human Judgment” (“Noise”), Kahneman and his colleagues, Oliver Sibony and Cass R. Sunstein, examine how different judges make sentencing decisions on a set of increasingly difficult legal cases^13^. They observe that noise is ever present and provide examples of how noise affects the way judgements or decisions are made in areas such as medicine, weather forecasting, and law. In order to study noise and consequently reduce it, they propose a *noise audit* that is intended to identify certain types of noise in situations where the same case is evaluated by many individuals. Among these types, *level* and *pattern* noise are identified as two especially important types that can be objectively measured, and that are found, in practice, to contribute significantly to *system* noise, which is the total variability in judgments across all cases and judges.

Before providing definitions or formulae for these types of noise, we provide some intuition by considering a simpler version of the example in “Noise.” Consider a set of *n* ‘judges’ and *m* binary decisions or ‘cases’ per judge e.g., a judge might be a magistrate who has to decide whether to grant bail (1) or not (0) to a defendant appearing before them. For the sake of simplicity, let us assume a hypothetical world where each of the *m* bail decisions is (independently) reviewed by *all* *n* magistrates. This exercise yields an $n \times m$ *judge-by-case matrix* with either 1 or 0 in each cell. In the absence of *interjudge disparity*, each column (representing the *m* decisions by the magistrates on a given defendant’s bail) would be a vector comprising either all 1’s or all 0’s. This is not to say that the *entire* matrix is either all 1’s or all 0’s; some columns could be all 0’s, while others could be all 1’s.

Given the judge-by-case matrix, the *level* of a judge is the mean value for that judge (or the row mean). In this case, if a judge has a higher level value than another judge, then it implies that they are more lenient (at least on average) in granting bail. Given the levels, the aggregate difference or disparity between judges can then be quantified by computing the standard deviation (SD) of the levels (in other words, the SD across row-means). The SD of the row-means is denoted in “Noise” as the *level noise*. Only if judges make identical decisions (and there is no difference between any two rows in the matrix), will level noise be zero; otherwise, by virtue of being a SD, it will be strictly positive. For this reason, Clancy et al.^23^, whose work is cited in “Noise” as a primary source, originally refer to level noise as *interjudge disparity* in their study of sentencing disparities among judges in the United States in the criminal justice system.

The intuition behind *pattern* noise is slightly more complex, as it is meant to explain why the variability in different judges’ levels alone is not enough to account for the overall variability in the matrix (defined as the *system* noise). By overall variability, we simply mean the SD computed over the list of *mn* numbers (obtained by ‘flattening’ the $n \times m$ matrix). One interpretation of pattern noise is that it is the residual noise that, together with level noise, explains the system noise. Because this interpretation defines pattern noise as a residual, its causes may be complex and dependent on factors that have nothing to do with either the case or the judge e.g., *occasion noise*, which occurs when (for example) judges are found to be statistically more lenient on days with good weather compared to days with bad weather^13,24^.

In the context of this study, however, it is more desirable to study the contribution to this residual that can be explained by the *prompts* in the benchmark. Taking the criminal justice example as an analogy, Kahneman et al. write that some judges “may be harsher than average in general but relatively more lenient toward white-collar criminals,” and with another source^25^ similarly referring to such phenomena as “patterned differences between judges” that arise because of the specifics of the case or defendant (such as a judge showing leniency to white-collar criminal defendants). Hence, a different way to interpret pattern noise is by defining it as the *variability among judges* on a case.

Continuing with the analogy above, if all judges sentence equally harshly on aggravated assault cases, but some judges are more lenient than others on white-collar criminal cases, the latter cases will exhibit inter-judge variability and will contribute non-trivially to pattern noise. When conducting a noise audit on human-labeled benchmarks, one advantage of adopting this definition of pattern noise is that it allows us to directly quantify the effects of the prompts in the benchmark on overall variability. Furthermore, because it is computed from first principles, similar to level noise, rather than *tautologically* (i.e., as the difference between system and level noise), it allows us to quantify how much of the residual is directly attributable to prompt-related patterns. Such effects can then be separated out of the residual, unlike other patterns (such as occasion noise) that are more difficult to tease apart from the residual, or (alternatively) control in experiments, especially in the online or crowdsourced setting where very limited information about annotators is available. We find empirically, however, that the contribution of the ‘remaining’ residual (after subtracting out the pattern noise from the original residual, as described above) is usually a small fraction of the original residual, bearing out the intuition in “Noise” that prompt-related (or case-related) effects constitute the largest component of the residual.

Next, we formalize some of the intuitions stated above. One additional detail to note before we do so is that the judge-by-case matrix can have “missing” values in some cells. If this is the case, we assume that aggregate statistics like averages and SDs in the treatment below are only computed over non-missing values. As long as no row or column in the matrix is completely empty, the key intuitions still apply. Based on the example above, we consider the human annotators as ‘judges,’ commonsense reasoning prompts as decisions or ‘cases,’ and annotators’ labels as the ‘judgments’ (decision outcomes). We maintain terminological consistency by assuming a single commonsense reasoning task $\mathbb {P} = \{P_1,\ldots , P_m\}$ that is comprised of a set of *m* prompts, and with a set $\mathbb {A} =\{A_1,\ldots , A_n\}$ of *n* annotators providing labels for the prompts. Without loss of generality, the labels can be assumed to be binary (0 or 1). In our experiments, as subsequently described in *Materials and Methods*, non-binary labels are binarized prior to noise analysis to facilitate comparisons using a unit scale. With this background in place, we define the following terms:

- $Labels(A_i)$ is defined as the (non-empty) set of all labels acquired from annotator $A_i$ across commonsense reasoning dataset $\mathbb {P}$.
- $Labels(P_j)$ is defined as the set of all labels acquired for prompt $P_j$ in dataset $\mathbb {P}$ (provided by some non-empty subset of annotators in $\mathbb {A}$ in the general case).

We note two important observations about both definitions. First, in keeping with the observation stated earlier that the judge-by-case matrix can have missing values, the definitions also don’t make the assumption that *every* annotator in $\mathbb {A}$ must have provided a label for *every* prompt in dataset $\mathbb {P}$, only that at least one label is available for each prompt (and also that every annotator must have provided at least one label to be included in $\mathbb {A}$). A special case of interest is when every annotator in $\mathbb {A}$ does provide a label for every prompt in $\mathbb {P}$, which also applied to the intuitive example provided earlier involving magistrates and bail decisions. This special case applies best to one of our datasets (TG-CSR), where there are few missing labels, and can usually be enforced in a lab setting, but not in typical crowdsourced online settings, where annotators are free to decide how many micro-tasks (in our case, prompts) they want to label and be paid for.

The second observation follows from the first, which is that the size of $Labels(A_i)$ is bounded above by the number (*m*) of prompts in $\mathbb {P}$, and is only equal to the bound in the special case mentioned earlier. For a given prompt, we assume that an annotator can provide at most one label. In the same vein, $Labels(P_j)$ is bounded above by *n*, the total number of annotators available in the pool $\mathbb {A}$.

Given these definitions, *level noise* (LN) in Kahneman’s noise framework can be formally defined as the SD among annotators’ *mean* labels (represented by the overline):

1 $$\begin{aligned} LN = SD(\{\overline{Labels(A_{1})},\ldots ,\overline{Labels(A_{n})}\}). \end{aligned}$$

Similarly, pattern noise (PN) can be defined as:

2 $$\begin{aligned} PN^{orig} = SD(\{\overline{Labels(P_{1})},\ldots ,\overline{Labels(P_{N})}\}). \end{aligned}$$

While this definition of pattern noise ($PN^{orig}$) measures prompt-specific effects, as we had described earlier, one issue with it in the specific context of our benchmarking noise audit is that its *ideal* value is not zero, and in fact, can be highly benchmark-specific. Consider, for example, a commonsense reasoning dataset where the prompt is a sentence, and the (binary) task is to label the sentence as plausible (label 1) or implausible (label 0). Suppose also that the dataset has 100 such prompts, and that there is perfect agreement among a large set of annotators that the first 60 prompts are plausible, while the last 40 are implausible. In this artificially idealized scenario, where the set of labels is as close to a true ground-truth as possible, *LN* is 0 (as expected), but $PN^{orig}$ is $\sqrt{(60 \times ((1-0.6)^2)+40 \times ((0-0.6)^2))/100} \approx 0.49$, which is far from 0. If now the fraction of plausible prompts in the dataset changes to 90 out of 100, and annotators still agree perfectly, *LN* remains 0, while $PN^{orig}$ now reduces to 0.3.

Interestingly, this situation can also arise in the criminal sentencing domain, and is mentioned as an issue by Clancey et al.^23^, but not in “Noise,” perhaps because it was considered too technical to include in a book meant for the general public. In fact, it is not difficult to see that this ‘anchoring’ issue (where the *ideal* pattern noise is not a priori anchored to an intuitive value like 0, unlike level noise) can arise in any task with an unknown ‘true’ distribution of class labels, and can lead to a misleading interpretation of the actual undesired noise in the system. The key issue is that $PN^{orig}$ depends on the distribution of those class labels in the underlying dataset. What is desired instead is a version of pattern noise that is ideally zero (similar to level noise). For completeness, we continue to report $PN^{orig}$ in our experimental results, but also propose a modified definition of pattern noise $PN^{mod}$ below that anchors the ideal pattern noise at zero for a given dataset:

3 $$\begin{aligned} PN^{mod} = SD\{SD(Labels(P_{1})),\ldots ,SD(Labels(P_{N}))\} \end{aligned}$$

The difference between $PN^{orig}$ and $PN^{mod}$ is that label means have now been replaced by label SDs. SD for a given *Labels*($P_j$) will only be zero if all annotators who provided labels for prompt $P_j$ agree. It is a measurement (albeit not the only one) of inter-rater disagreement on a given prompt. In principle, SD is not qualitatively dissimilar to other such measures, such as Cohen’s kappa^26^, although we caution against equating or co-substituting the two in subsequent analyses. $PN^{mod}$ should instead be interpreted as a ‘benchmark-normalized’ version of $PN^{orig}$ that enables appropriate comparisons *across* benchmarks, without requiring us to control for the distributions of labels within those benchmarks. It is true to the spirit of the pattern noise that we intuitively described earlier: to quantify noise that arises as a consequence of “patterned differences” between annotators because of the prompts. A subtle point to note is that the outer SD further ensures that noise is computed over such “*patterned* differences” (emphases ours) and not just an average over disagreements on individual prompts i.e., in an extreme case, it is theoretically possible for the inner SDs to be non-zero but largely similar, which expresses high average disagreement, but would still lead to a low value for the pattern noise as a whole because the pattern of disagreement is uniform across the prompts. In such a situation, assuming overall variability (system noise) is high, the pattern noise would be dominated by the remainder of the residual.

In practice, an argument can be made that $PN^{mod}$ itself is associated with a measure like Cohen’s kappa, but even if true, an important difference that we emphasize here (and that is an advantage of using the “Noise” framework) is that both the LN and PN definitions use compatible scales, meaning that the overall variability, or system noise (SN), can be decomposed using the following formula:

4 $$\begin{aligned} ({{SN}^{orig}})^2 = {LN}^2 + ({PN^{orig}})^2 + Residual \end{aligned}$$

Note that SN can be computed empirically from the judge-by-case matrix as the ‘overall’ SD over the set of all non-missing values in the matrix. If there is no *statistical interaction* effect between the judges and cases, the residual in the equation above will be zero. In “Noise,” the residual was folded into the (original) pattern noise, but here we choose to report both separately. We argue that this provides greater insight, both because we have defined two different versions of the pattern noise (original and modified), allowing us to compare the two, and also because of the prompt-specific interpretation of the pattern noise (without the residual folded in) in the benchmarking context being studied here.

System noise in Eq. (4) (${SN}^{orig}$) suffers from the same problem as ${PN}^{orig}$; namely, it expresses the total variability, but not all of this variability is *unwanted*. As we showed earlier, ${PN}^{orig}$ depends on the distribution of labels in the benchmark, which can lead to benchmark-specific estimates of pattern noise. At the same time, ${SN}^{orig}$ is still a useful reference because it shows the total variability in the judge-by-case matrix. One way to calculate the total *unwanted* variability is by defining a modified version of system noise (${SN}^{mod}$) as follows:

5 $$\begin{aligned} ({{SN}^{mod}})^2 = {LN}^2 + ({PN^{mod}})^2 + Residual \end{aligned}$$

A subtle, but important, point to note here is that the *Residual* is calculated using Eq. (4) by rearranging the terms (since, as mentioned earlier, $SN^{orig}$ can be directly computed as the SD over all non-missing values in the judge-by-case matrix), but is then plugged into Eq. (5) to express unwanted variability as the sum of three different components. With this background in place, we define a *noise audit* on dataset $\mathbb {P}$ as a tuple of six variables: $(LN, PN^{orig}, PN^{mod}, SN^{orig}, SN^{mod}, Residual)$.

## Materials and methods

### Data

To investigate RQ1 in a lab-based homogeneous setting, we used the machine commonsense benchmark *Theoretically-Grounded Commonsense Reasoning*^16^ (TG-CSR), which consists of eight datasets for the following four contexts: *vacationing abroad, camping, bad weather*, and *dental cleaning*. The vacationing abroad context is about a person named Chloe who is planning to take a vacation abroad with some friends. The camping context is about a couple who are planning a camping trip to the White Mountains in New Hampshire. The bad weather context references examples of bad weather situations, and the prompts question how a reasonable person should respond to a particular bad weather situation, e.g., a hurricane. The dental cleaning context contains prompts centered on a meeting with a dental hygienist for a standard dental cleaning procedure. TG-CSR was intentionally built around contexts with prompts that we assume can be answered with commonsense knowledge.

The annotators were provided with the data in two formats. One format presents the prompts as multiple-choice sentences. For each of 9 fundamental commonsense reasoning categories^27^ (time, goals, scheduling, world states, activities, emotions, physical entities, space and values), 2–5 prompts are provided, along with a list of approximately 10 answer options^28^. The second format presents each of the multiple-choice prompts as a *true–false* (T–F) question. Table 1 provides examples of multiple-choice and T–F prompts for each category in the camping context. The multiple-choice prompt column in the table displays a single multiple-choice prompt, the next column displays several answer options, and the last column displays a prompt associated with one of the multiple-choice answer options in a T–F format.

The data contains a total of 2379 prompts: 331 prompts for vacationing abroad multiple-choice, 326 prompts for vacationing abroad true-false; 340 prompts for camping multiple-choice, 340 for camping true-false; 297 prompts for bad weather multiple-choice, 297 prompts for bad weather true-false; 224 prompts for dental cleaning multiple-choice, and 224 prompts for dental cleaning true-false. Note that the number of prompts in the T–F version of the vacationing abroad dataset is lower than the number of prompts in the multiple-choice format because 5 of the prompts (4 associated with the “goals” category, and 1 associated with the “physical entity” category) were considered to be too ambiguous when converted into a T–F format, and therefore removed.

**Table 1:** Data from TG-CSR’s *camping* context. The top part of the table shows the context and theme. The bottom part provides two example prompts for each of the nine common sense categories: one prompt for the multiple-choice format (with example answer options separated by commas), and one corresponding prompt in the true–false (T–F) format. For the multiple-choice modality, each answer option is placed in its own cell in a spreadsheet, and an annotator has to enter a rating from 1 to 4 (explained in “Annotation Methodology”) for each option independently.

Context	Camping vacation		
Theme	Fred and Linda want to take a vacation in the White Mountains of New Hampshire in August. They want to spend about a week doing day hikes and stay in a camp ground that is close to the trails that they like. Fred went on a few camping trips as a child but Linda has never been camping. Fred’s brother has offered to loan him a small tent and some camping equipment		

Category	Multiple-choice prompt	Answer options	True/false prompt
Time	How long might it take to set up a small tent?	1 h, 9 days, 5 min	It takes 1 h to set up a small tent
Goals	What are some common reasons for taking a camping vacation?	Watching wildlife, It is inexpensive, Find a good spot for the tent	Watching wildlife is a common reason for taking a camping vacation
Scheduling	What activity might Fred and Linda plan to do on their first day in the White Mountains?	Take a 5-mile hike, Have breakfast at a local restaurant, Drive home	On their first day in the White Mountains Fred and Linda plan to take a 5-mile hike
World States	Linda and Fred had to quickly leave the campground. What caused them to leave?	They heard that the main highway was going to close, A car hit and knocked over a trash can, There was a rain shower	Linda and Fred had to quickly leave the campground because they heard that the main highway was going to close
Activities	While camping, what activities are best to do before it gets dark?	Gather firewood, Cook, Gaze at stars	While camping, an activity that is best to do before it gets dark is to gather firewood
Emotions	How will Linda feel if she encounters a bear at their campsite?	Fear, Upset, Happy	If Linda encounters a bear at their campsite, she will feel happy
Physical Entities	What items might Fred and Linda carry with them when they take a day hike?	Water bottle, Mosquito repellent, A cooler	Fred and Linda might carry a water bottle with them when they take a day hike
Space	When you are hiking, where is the best place to carry your water bottle?	In a backpack, In a coat pocket , 1000 feet	When you are hiking, the best place to carry your water bottle is in a backpack
Values	How many people can sleep in a small tent?	2, 1, 9	The number of people that can sleep in a small tent is 2

These eight datasets were annotated by six human annotators using the methodology described in the next section. The noise audits and metrics underlying both RQ1 and RQ2 use the complete set of labels acquired across these eight datasets, as subsequently detailed.

To investigate RQ1 in the more realistic crowdsourced setting, we used the SemEval-2020 Commonsense Validation and Explanation (ComVE) Challenge dataset^29^. This dataset serves as an evaluation benchmark that enables us to directly assess a system’s ability to distinguish between plausible natural language statements and those that are not. The dataset comprises three subtasks, with our primary emphasis being on the first subtask (subtask A). In subtask A, a system is tasked with selecting the more plausible statement from a pair of natural language statements that have similar wording, with one being plausible and the other (relatively) implausible. An example of a statement pair from the ComVE subtask is “He drinks milk.” and “He drinks apple.” The implausible nature of the latter statement is apparent, as apples cannot be drunk, while the former statement is clearly more plausible. Unlike TG-CSR, where all labels provided by the six (anomymized) annotators are made available, the ComVE benchmark was only accompanied by a single set of ‘gold standard’ labels, the provenance of which is not completely evident in the documentation of the benchmark. Hence, to conduct a proper noise audit of ComVE, all 2000 sentences within it (from 1000 statement pairs from the training dataset in ComVE subtask A) needed to be ‘re-annotated.’ The methodology for doing so is described in the next section.

Concerning performance of the language models on ComVE, even prior to the emergence of generative large language models, such as the GPT models, discriminative language models such as BERT and RoBERTa achieved impressive performance on ComVE (with accuracy exceeding 0.8 on both the development and test partitions^30^). Nevertheless, it is possible to improve performance even further by a few percentage points. For example, employing ensemble models that utilized majority voting among various BERT-based models was found to yield even higher accuracy rates surpassing 0.9 on both development and test sets. In contrast, to our knowledge, even the large language models have been unable to achieve accuracy higher than 0.8 on the TG-CSR dataset, although performance has still been reasonably good (0.6 or higher). This is not unexpected, since TG-CSR had been designed to be a more comprehensive and challenging commonsense reasoning dataset compared to earlier benchmarks like ComVE. We also investigated the performance of a large language model (ChatGPT) when applied to ComVE, and show how the estimate of this performance can change when a full set of independent human labels are considered as the reference, rather than a single unambiguous ground-truth.

### Annotation methodology

The prompts within the TG-CSR benchmark used for the experiments in this article were split into spreadsheets (Excel files with tabs), and provided to six annotators, along with instructions and general considerations to keep in mind. For each of the four contexts, we created a true-false and a multiple-choice formatted spreadsheet, yielding eight spreadsheets in total. Prompts corresponding to the nine commonsense categories stated earlier were placed in different tabs in the spreadsheet; however, we used opaque names for each tab to avoid priming or biasing the annotator in any way. The annotators were all affiliated with the two organizations conducting the experiment. Three of the annotators were PhD students^31^ and three were researchers involved in this study. All annotators took 15–35 min to complete the annotation of all of the prompts in a single dataset. They completed the annotations independently, in their own work environments. To ensure that the annotation is as unbiased as possible, there was no incentive to maintain a specific distribution of labels (e.g., 50% True and 50% False); furthermore, TG-CSR was never used to evaluate, either pre- or post-annotation, any commonsense reasoning system that any of its authors developed.

**Figure 1:**
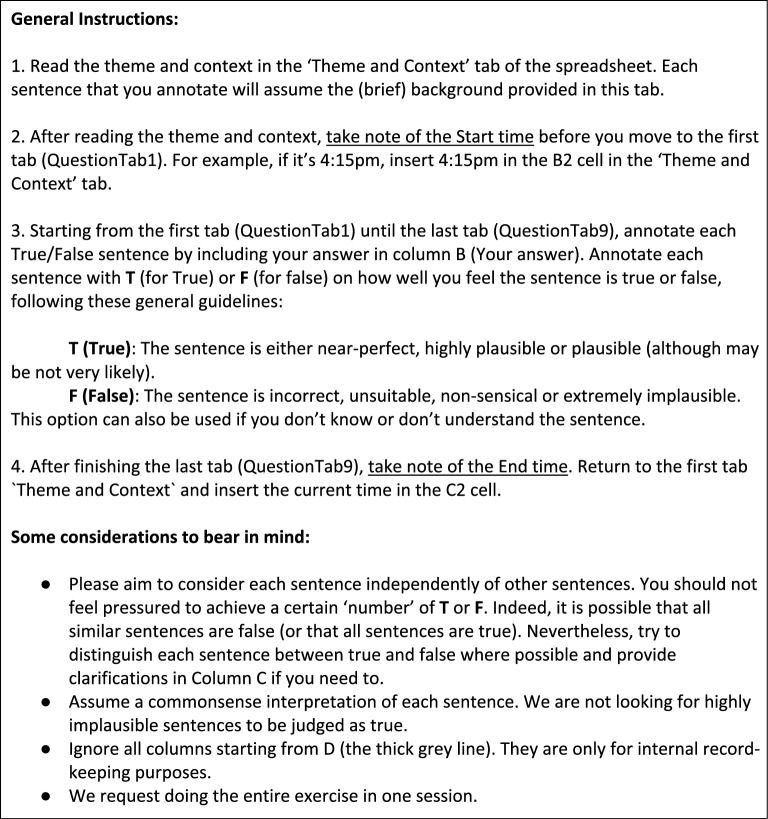
Annotation instructions presented to human annotators for the True/False format of the TG-CSR dataset.

In Fig. 1, we reproduce the exact instructions provided to the annotators (for the True/False format) in a separate document before they opened the spreadsheet and started annotating. As shown therein, the instructions recommended responding to all of the prompts in a given dataset in one session, providing the start-time and the end-time of their session in the *Theme* and *Context* tab, and reading the content of the *Theme* and *Context* tab before beginning to answer the prompts.

For the multiple-choice formatted prompts, the instructions were similar except that the annotators were instructed to rate the appropriateness of each of the provided answer options given a prompt, from a human commonsense perspective, using one of the following four labels: *4 (very good fit), 3 (good fit), 2 (not sure), 1 (bad fit)*. In contrast, the instructions for the T–F formatted prompts requested the annotators to respond to each prompt using either T (True) or F (False) (note from the examples in Table 1 that each answer option was ‘folded’ into the original prompt to create a *new* prompt for which a true or false answer made sense).

To obtain responses to each of the 2,000 prompts in the ComVE benchmark, we used Amazon Mechanical Turk (MTurk)^32^. MTurk is a popular crowd-sourcing platform that enables businesses and individuals to outsource a wide range of tasks to a global workforce. The platform offers a diverse range of tasks that can be completed remotely, including data validation, survey participation, and more. For completing each such *Human Intelligence Task* (HIT), a fee is paid to the ‘turker’ (who is the annotator in this experiment). Turkers can select from a pool of available HITs on the platform and earn money by completing them. MTurk has been used in various fields, including both social science and computer science (especially for constructing ground-truths at a reasonable cost and quality)^33^. It provides access to a large and diverse pool of workers who can complete tasks quickly and efficiently.

To re-annotate ComVE, we modeled our HIT as rating a randomly-selected sentence from the ComVE corpus of 2000 sentences as plausible or implausible, and required that each sentence be rated independently by at least five turkers. Internally, we encoded the plausible rating as ’1’ and implausible rating as ’0’ for subsequent analysis. A total of 244 turkers participated in the annotation task, and a total of 10,000 unique labels were obtained. Note that we provided the crowdsourced workers with one independent statement each time and required them to assess if the statement is plausible or not without any knowledge of its pairing with another statement (to avoid issues of priming). An annotator was not limited in the number of labels they could provide, but each label had to be for a unique sentence. In other words, while a turker can provide a label for more than one prompt, the same turker was never ‘re-presented’ with a prompt once they provided a label for that prompt. Furthermore, once the five-annotator limit had been reached for a sentence, the platform would automatically exclude the sentence from further annotation. On average, the metadata showed that each worker annotated 40.98 statements. The annotator who evaluated the most statements annotated 258 in total. We comment more on the statistical distribution of the number of labels acquired per annotator, and its connection to the observed levels of noise in the labels, when discussing the results for RQ1.

### Noise audit and analysis

To compare noise in the multiple-choice format with the T–F format, and to facilitate the comparison of the TG-CSR data with data from ComVE, we ‘binarized’ the raw labels acquired from annotators in the multiple-choice format (using the 1–4 scale) by encoding the (original) 1 and 2 labels as 0, and the (original) 3 and 4 labels as 1. As part of this preprocessing, we also removed missing or invalid (i.e., a number not within 1–4) labels from the data. The code considers them as *null* during computation, and ignores them when computing standard deviations, averages, or any other relevant aggregate measures. The prevalence of such invalid or missing labels was low (fewer than ten instances per annotator in any given TG-CSR context). All labeled data was converted to a machine readable JavaScript Object Notation (JSON) format to support analysis, and is made available with the manuscript to facilitate replication and other studies.

As described earlier in Eq. (1) in *Noise Audit Framework*, level noise is defined as the standard deviation among annotators’ labels, or more intuitively, the variability of the average judgements made by different individuals. For RQ1, we apply Eq. (1) across all datasets to compute individual level noises. In contrast, pattern noise is attributed (in Kahneman’s work) to an individual’s bias and cognitive style. In our specific context, pattern noise similarly accounts for the discrepancy among annotators’ labels for the *same* prompt. For reasons detailed before, we compute two versions of pattern noise, using Eqs. (2) and (3), denoted as the *original* ($PN^{orig}$) and *modified* ($PN^{mod}$) pattern noise, respectively. We compute the system noise and residual as discussed toward the end of *Noise Audit Framework*. As noted therein, $SN^{orig}$ is directly computed from the judge-by-case (annotator-by-prompt) matrix, following which the residual can be computed by rearranging the terms in Eq. (4). By plugging in the residual in Eq. (5), we obtain a measure of the ‘unwanted’ variability in the system as $SN^{mod}$.

Conducting a noise audit of ComVE was more challenging, as the set of labels corresponding to all prompts in ComVE is more heterogeneous and because turkers will only provide labels for a variable subset of prompts. ComVE has 2000 prompts, and in an ideal experiment, every turker would provide a label for every prompt. However, even if it were not cost prohibitive to do so, turkers cannot be forced into answering all 2000 prompts (i.e., an individual turker can choose how many micro-tasks to accept and get paid for). This implies that many (let alone, all) prompts are not annotated by a *common* turker; nor is it the case that any two prompts are necessarily annotated by the same set of five turkers. This leads to worker heterogeneity, and consequently, more noise might be expected due to greater individual differences. However, it was always the case that exactly five individual labels were acquired for each prompt, because of the manner in which the micro-task was set up in the MTurk platform. Other than this heterogeneity, the ComVE re-annotation was not dissimilar from the annotation of the T–F version of TG-CSR in that each prompt in the latter was labeled (excepting for the few instances with missing or syntactically invalid labels, which we discarded in the analysis) by six annotators, and in the former by five (varying) annotators.

#### Effect of filtering on noise

Because there is considerable variance in how many total labels were provided per annotator, one of our secondary goals in conducting a noise audit on ComVE using crowdsourcing, is to analyze the *changes* in different types of noise levels when we apply filters or ‘cutoff’ methods by only considering annotators who provided either a minimum, or a maximum, number of labels. For example, we may wish to remove annotators in our analysis who did not provide at least ten total labels. This kind of filtering is commonly applied in crowdsourcing tasks, mainly as a heuristic method for quality control^34^. In this paper, we instead treat the question as an open empirical one: should annotators in an online setting be filtered ‘out’ based on the numbers of labels provided? In theory, there is no reason for the number of labels provided to be correlated with the quality of the labels (measured in terms of noise).

Within this noise framework, we would expect such quality problems to manifest in changing levels of aggregate noise once we apply exclusionary filters on annotators, based on how many labels they provided. For example, by setting a minimum allowable label limit (that we vary as an independent variable in our analysis) per worker, we can determine if more “experienced” annotators resulted in lower noise levels. Similarly, we can toggle a *maximum* allowable label limit per worker to assess if excessive labeling effort could lead to higher noise levels (e.g., due to hypothetical reasons such as cognitive fatigue). We actively study the results of such filtering on noise in the results. However, we note again that, except when we are explicitly studying the effects of filtering, all annotators and their labels are always used in any analysis involving ComVE i.e., no annotator is precluded *a priori* from an analysis because of the numbers of labels provided (even if only one label was provided).

One additional point to note is that applying such filters is not without cost. An aggressive filter could lead to many annotators (and their labels) getting removed, leading to fewer prompts remaining in the dataset to be analyzed. For example, when we set the minimum allowable labels for each turker to 10, we find that there are 181 turkers (from the original 244) who provided labels for at least 10 statements in the dataset. A second issue that arises is that each prompt is now not guaranteed to have a total of five labels. We still consider each prompt in our analysis as long as at least two labels were retained for the prompt. However, when describing the results, we also provide distributional data on how many sentences were retained (after applying a filter) with no labels, exactly one label, two labels, and so on.

#### Performance analysis of humans as commonsense reasoning systems

While RQ1 aims to investigate overall levels of noise across TG-CSR and ComVE through a noise audit, the motivation behind RQ2 is to understand how noise can affect the reliability of a ‘ground-truth’ derived using a set of annotations. Such ground-truths are generally used to evaluate supervised machine learning systems, including large language models. Many commonsense reasoning benchmarks in the natural language processing community are structured similarly as TG-CSR^4^: as question-answering instances with a closed set of provided choices (whether in a multiple-choice format, or as *True* and *False*). To quantify how noisy such *putative* ground-truths can be, RQ2 treats each annotator as an independent ‘system’, while the other annotators are used to compile the ground-truth against which the ‘system’ is evaluated (by computing its accuracy)^35^. Specifically, this ground-truth is constructed using the mode of the labels provided for that prompt by the other annotators.

We illustrate this as follows. Let us denote the six annotators used for the TG-CSR experiments using the (fixed) symbols A, B, C, D, E, and F. If we are evaluating A as the ‘system’, we take the mode of the labels provided by B, C, D, E, and F as the ground-truth label for a given prompt. Hence, if there are X prompts in the dataset, the per-prompt mode will yield a ground-truth with X labels (one for each prompt). Similarly, A has provided a label (not taken into account in the mode) for each of the X prompts. Within a given prompt, if A’s label then matches the modal (ground-truth) label exactly, it is considered ‘correct’ (1), otherwise ‘incorrect’ (0). A’s ‘performance’ on a dataset is then represented as a vector of 1’s and 0’s. Using this vector of 1’s and 0’s, A’s accuracy can be estimated as the mean of this vector, along with a 95% confidence interval of the estimate (using the z-test, since there are well over a hundred prompts even in the smallest of the four contexts within TG-CSR).

We can compute a similar accuracy estimate, with a 95% confidence interval, for annotator B (by taking the mode of the labels of A, C, D, E, and F for each prompt), and so on, for C, D, E, and F. Furthermore, the same calculation can be repeated for each of the eight TG-CSR datasets (spanning two formats and four contexts). We report and compare all of these estimates to investigate RQ2.

We conducted a similar performance analysis for ComVE. First, to enable comparison to TG-CSR in the same vein as the noise audit comparisons between the two benchmarks, we compute an accuracy estimate (of human performance on ComVE) by again using a leave-one-annotator-out strategy, and comparing that annotator’s label to a ‘reference’ ground-truth computed as the mode of the labels of the remaining annotators for a given prompt. However, unlike TG-CSR, the leave-one-annotator-out strategy will not work for *all* prompts due to the annotator-heterogeneity in ComVE (described earlier).

To mitigate this issue, we first consider all annotators who have provided a minimum number of labels *n* (with *n* being an experimental parameter that we vary, from 0 to the maximum number of labels provided by any turker, when reporting results; note that, when *n* equals 0, *all* annotators and labels are accounted for). By toggling *n*, we filter out annotators who have not provided enough labels only for computing that specific data point. We illustrate this more formally as follows. For a given value of *n*, suppose that we denote *A*(*n*) as the set of annotators who have provided at least *n* labels. Next, for an annotator $a \in A(n)$, we consider all prompts that *a* has provided a label for. For each of those prompts, the ground-truth label is set to be the mode of the labels of the four remaining annotators that have provided labels for that prompt (and who may not necessarily be in *A*(*n*)). For fairness, we do not consider prompts where the four annotators ‘split’ on their labels (with two rating the sentence as plausible, and two rating it as implausible). In this way, we can compute a deterministic accuracy estimate for *a*, and in a similar vein, for *every* annotator in *A*(*n*). This allows us to plot a distribution of accuracy estimates (e.g., as a box plot) for each value of *n*. Note that in the special case $n=0$, all annotators would be included in *A*, and hence, we obtain a distribution that can be directly compared to the distribution described earlier for TG-CSR. Additionally, we obtain distributions for other values of *n*, which allows us to assess the effect of filtering on the variability observed for each distribution. Finally, we use the same data to plot a distribution similar to the one described above for TG-CSR, but with $n \ge 60$ to ensure that confidence intervals can be computed with sufficient power using the normal distribution.

#### Performance analysis of ChatGPT on ComVE

We also conducted a performance analysis of ComVE using ChatGPT as a commonsense reasoning system, rather than an actual human. Since its release, ChatGPT has yielded remarkable performance improvements on several AI problem domains, especially involving natural language understanding^36^. To understand how the performance estimate of ChatGPT can change in the presence of noise, we first randomly sample 100 sentences in each of three categories (obtaining a total of 300 sentences): **(i) no noise:** these are the sentences where all annotators had perfect agreement i.e., either all annotators thought the sentence was plausible, or all thought it was implausible; **(ii) moderate noise:** these are the sentences where one out of five annotators disagreed with the other four; **(iii) noise:** these are the sentences where two annotators disagree with the other three. Note that of the 2000 sentences that were annotated using crowdsourcing, 1249 sentences had no noise, 497 sentences had moderate noise, and 254 sentences were noisy. The three categories form a partition, as no other combination is possible beyond the ones listed above.

We sampled 100 sentences from each of these bigger ‘populations’ as OpenAI charges for each invocation of ChatGPT using the API. Hence, we chose to only conduct the ChatGPT experiments on a sufficiently sized sample that would allow us to use the Z-test to evaluate statistical significance. Once the sentences were obtained, we compute an estimate of ChatGPT’s performance on each of the three categories, using the mode of all five labels of a prompt to serve as a reference. Recall, however, that ComVE was released with a putative ground-truth. Therefore, along with using the mode of the labels that we collected as primary data, we also use the putative ground-truth (for each of the three 100-sentences sets) as a reference. By comparing the performance of ChatGPT on the three sets, using either version of ground-truth, we can quantify how noise affects performance estimates.

## Results

### RQ1: Conducting a noise audit of commonsense reasoning benchmarks

Table 2 contains results from the noise audit that we conducted over the eight-dataset (four contexts and two formats) TG-CSR benchmark for investigating RQ1. Level noise is found to be consistently non-zero in all datasets, although (excepting a small increase in *Vacationing Abroad*), it is lower in the TF format than in the corresponding multiple-choice format. We note that this finding is aligned with Kahneman’s expectations in his book: an easier-to-understand (and less ambiguous) scale is likely to produce less noise. Recall that the original scale provided to annotators in the multiple-choice format was a 1–4 scale that was subsequently binarized to facilitate comparisons with the TF format. Despite the binarization, level noise remains higher in the multiple-choice format compared to the TF format (often by relative margins of more than 50%). Attesting to the commonsense nature of the datasets, we do not observe meaningful differences across the four contexts.

**Table 2:** A summary of the level, pattern and system noise across each of the four contexts and two formats (total of eight datasets) that compose TG-CSR. The top part of the table shows level, pattern, and system noises, as well as the residual, for the multiple-choice format. The bottom part shows noises for the true–false (T–F) format. We use both definitions of pattern noise ($PN^{orig}$ / $PN^{mod}$), and system noise ($SN^{orig}$ / $SN^{mod}$), introduced earlier in *Noise Audit Framework*. Recall that $SN^{orig}$ is calculated directly from the annotator-by-prompt (also called the judge-by-case) matrix, while $SN^{mod}$ is calculated using Eq. (5) by plugging in the residual obtained by rearranging the terms in Eq. (4).

	Vacationing abroad	Camping vacation	Bad weather	Dental cleaning
Multiple-choice format				
Level noise	0.0354	0.0636	0.1619	0.0660
Pattern noise	0.3804/0.2241	0.4032/0.2249	0.3985/0.2340	0.4005/0.2303
System noise	0.4571/0.3382	0.4749/0.3369	0.4818/0.3579	0.4876/0.3611
Residual	0.0629	0.0589	0.0471	0.0730
True/false format				
Level noise	0.0462	0.0205	0.0508	0.0242
Pattern noise	0.4013/0.2291	0.3967/0.2223	0.4138/0.2229	0.4099/0.2282
System noise	0.4756/0.3430	0.4672/0.3322	0.4839/0.3355	0.4872/0.3485
Residual	0.0633	0.0605	0.0603	0.0688

In contrast, a clear pattern is not observed across the pattern and system noise levels between the two formats, regardless of whether we use the original or modified versions of the definition. For *Bad Weather* and *Dental Cleaning*, we do see a small decrease for the modifier versions, but not by the same margins seen earlier with level noise. The noise is significantly higher than zero for the modified version, which was defined to be anchored at zero in the ideal situation (unlike the original version, which can be non-zero even when all annotators agree on all labels). As expected, the system noise is dominated by the pattern noise, with the residual playing a smaller, but still non-trivial role, especially in comparison to the level noise. Hence, the data does suggest the presence of other additional sources of noise besides the pattern noise and the level noise, and with the net effect of these other sources being roughly equal to the level noise.

The high levels of pattern noise suggest that even on a fixed or given prompt, annotators can disagree on their labels. Hence, some prompts might be more ‘ambiguous’ or difficult than others. However, there are also many prompts where there is minimal, or zero, disagreement among annotators (modified pattern noise is zero). When evaluating AI systems, it may be worthwhile separating the difficult prompts from the ones where perfect agreement among humans was achieved; however, in current benchmarks, this is impossible due to only a single ground-truth being available, with little additional data available about which prompts humans tended to disagree on.

**Figure 2:**
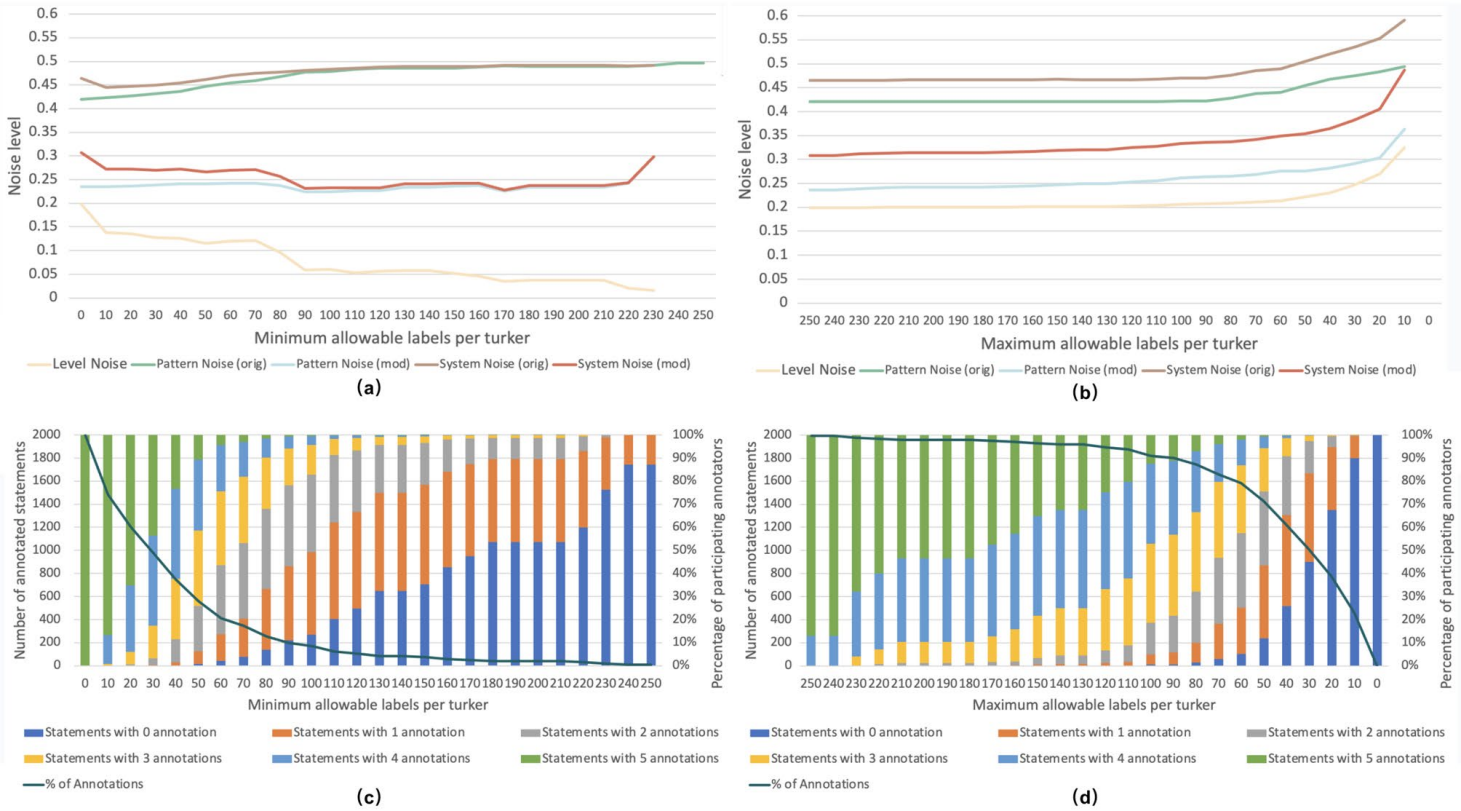
The noise level observed in ComVE annotations when controlling the minimum (**a**) and maximum (**b**) allowable labels for each turker (we omit the residual in these two figures as, similar to what was observed earlier for TG-CSR, it was nearly coincidental with the level noise curve). We also report the statistics of statements categorized by the number of annotation labels under different cutoff methods used for screening participating annotators in (**c**) and (**d**).

Figure 2 summarizes the results for ComVE. Specifically, Fig. 2a,b present the noise levels for the ComVE annotations while filtering by the minimum and the maximum allowable labels per worker, respectively. The figures show that system noise is again dominated by pattern noise (regardless of whether we are considering the original or the modified version), and that the noise levels observed are similar to those observed earlier for TG-CSR. Considering the first filter (minimum allowable labels per worker) level noise starts higher when we do not conduct any filtering, but declines in steps and eventually dips below 5% once a sufficiently high minimum has been imposed. In contrast, we do not see change in noise levels when imposing the maximum filter.

Figure 2c,d provide an estimate of the ‘cost’ of doing such filtering by showing how the percentage of annotations (or labels) declines in a non-linear fashion as the filter ‘threshold’ is made more aggressive. The color-coded bars also show that, while the number of statements with five labels declines quite quickly with an increasing threshold, the number of statements with at least two labels is still around 75% of the original, even when the threshold (Fig. 2c) is as high as 80. It is worth noting that, even when level noise is reduced due to this filter, pattern and system noise both remain relatively constant throughout and the level of this noise is similar to that observed for TG-CSR. Based on the definition of pattern noise, the same conclusion holds: some fraction of the statements are difficult or controversial, at least for humans. This difference between prompts is not reflected in the single ground-truth released as part of the ComVE dataset.

### RQ2: Effect on performance estimates of commonsense reasoning systems due to noise

**Figure 3:**
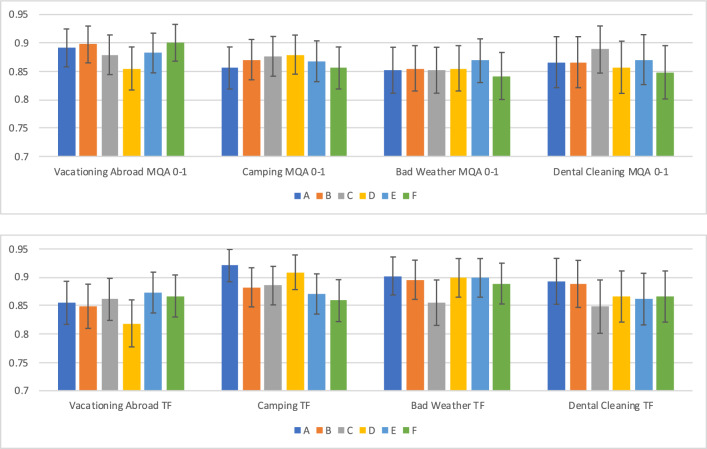
Accuracy estimates, with 95% confidence intervals, for each annotator (denoted using the fixed symbols A–F) on each of the eight TG-CSR datasets (separated by modality and context). The bars show accuracy scores when one annotator (specified in a color) is considered to be a ‘system’, while the mode of the labels from the remaining five annotators is used to construct a ‘ground truth’ for the set of prompts over which the the accuracy estimate of the ‘system’ is computed.

**Figure 4:**
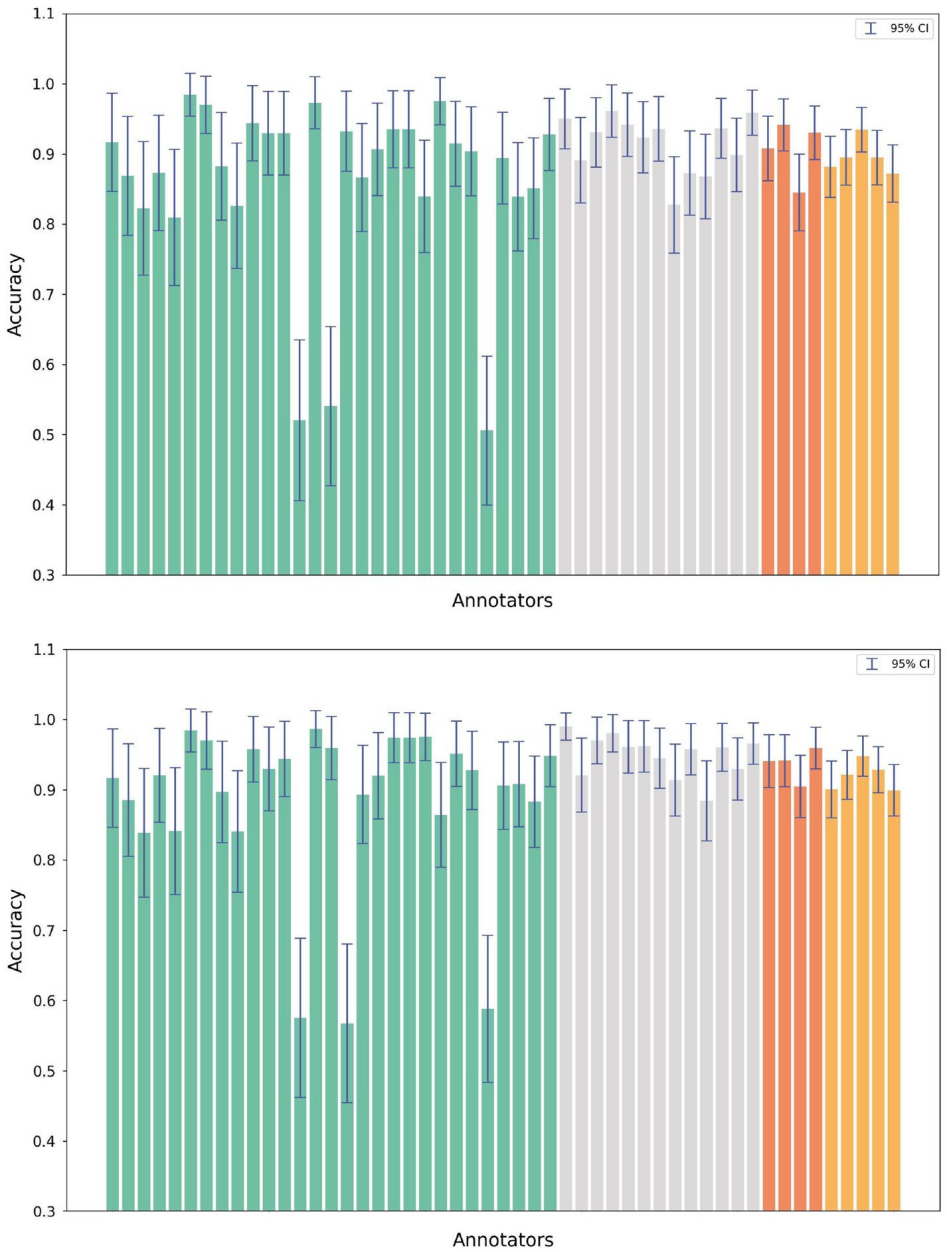
Accuracy estimates, with 95% confidence intervals, for the ComVE dataset. Each bar represents a turker. The colored bars represent the accuracy obtained when the annotations from a single turker serve as the ‘system’ predictions. We consider two different definitions of the ground-truth against which the ‘system’ is evaluated: (top) the mode of the remaining four turkers (for a fixed prompt); (bottom) the externally constructed ground-truth that was released with the original ComVE dataset. The arrangement of the bars corresponds to the number of annotations contributed by each turker. Light green bars indicate turkers who contributed 60–99 annotations, grey for those with 100–149, orange for 150–199, and yellow for those exceeding 200 annotations. For this plot, we do not consider turkers who provided fewer than 60 annotations due to insufficient power in constructing a statistically valid 95% confidence interval.

Figure 3 summarizes the findings for RQ2 when using the TG-CSR benchmark. As expected, accuracy is generally high (typically above 85%), since we are measuring human performance on commonsense reasoning tasks. The estimates across contexts are reasonably consistent (in line with the consistency noted across contexts in the results for RQ1), and also commensurate, especially when we take into account the standard errors, with human-level performance noted across most external commonsense reasoning benchmarks, such as PIQA^37^, HellaSwag^38^ and SocialIQA^9^. Nevertheless, the 95% confidence intervals (with half-widths of the bars in Fig. 3) of almost 3–5% in most of the accuracy estimates demonstrate the practical role that noise can play when evaluating systems on commonsense reasoning benchmarks. These confidence intervals are especially of concern because, in many documented cases of benchmark construction, only one or two annotators are typically used for acquiring ‘ground-truth’ labels. Indeed, what the intervals suggest is that, in at least a few QA instances, there is disagreement among annotators on the label, and this causes statistical fluctuation across accuracy estimates. In other words, even if we have a human-level AI system labeling QA instances across a benchmark, and we compare it to a baseline system, a difference of 3–5% does not *necessarily* mean that the former system is better than the latter: any such difference could also be explained (at least partially) by the underlying variance across annotators. We comment more on this variance and the limits that it might impose on (for example) statistical significance comparisons between two systems in the “Limitations and Implications” section.

**Figure 5:**
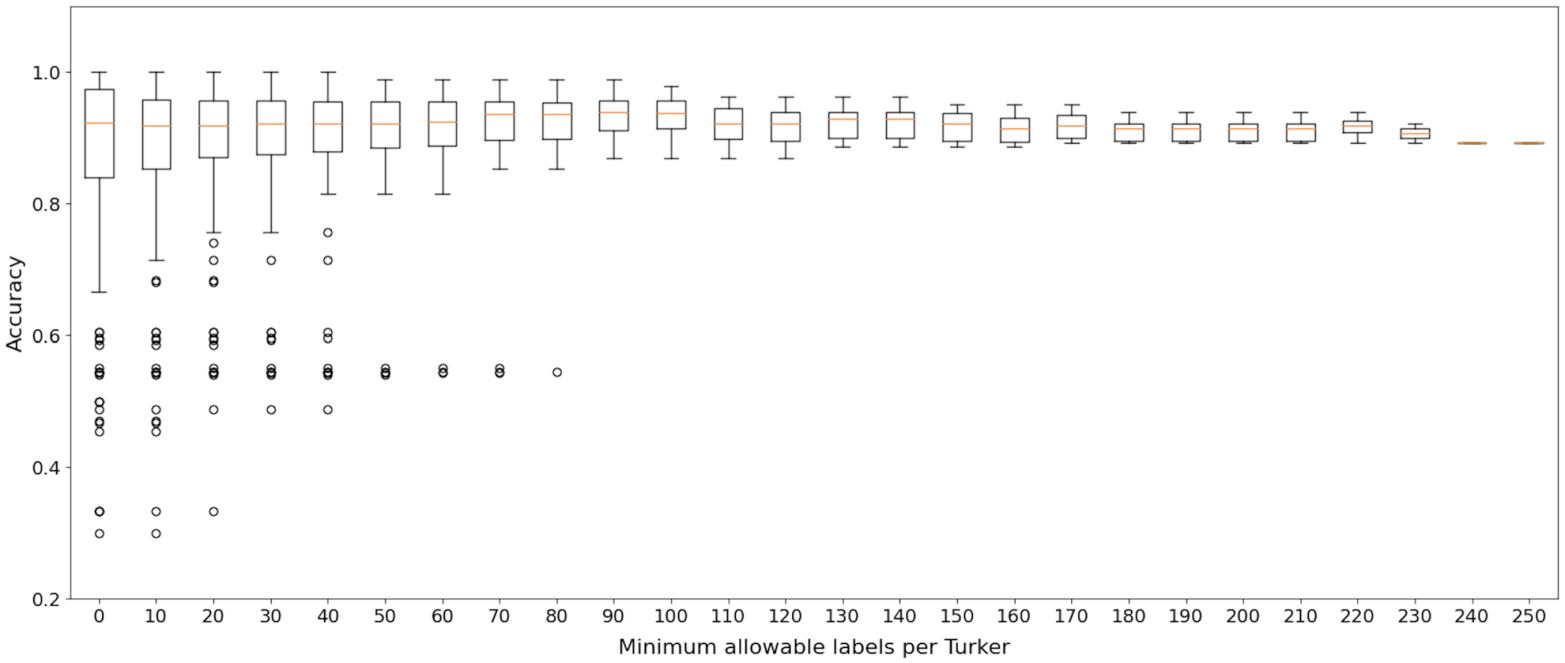
The annotation accuracy of turkers (shown as boxplots) on the ComVE dataset when filtering using the minimum allowable labels per turker as the criterion, with the ground-truth label of each prompt computed as the mode of the labels of the (four) remaining annotators who provided labels for that prompt, as described in *Noise Audit and Analysis*.

**Figure 6:**
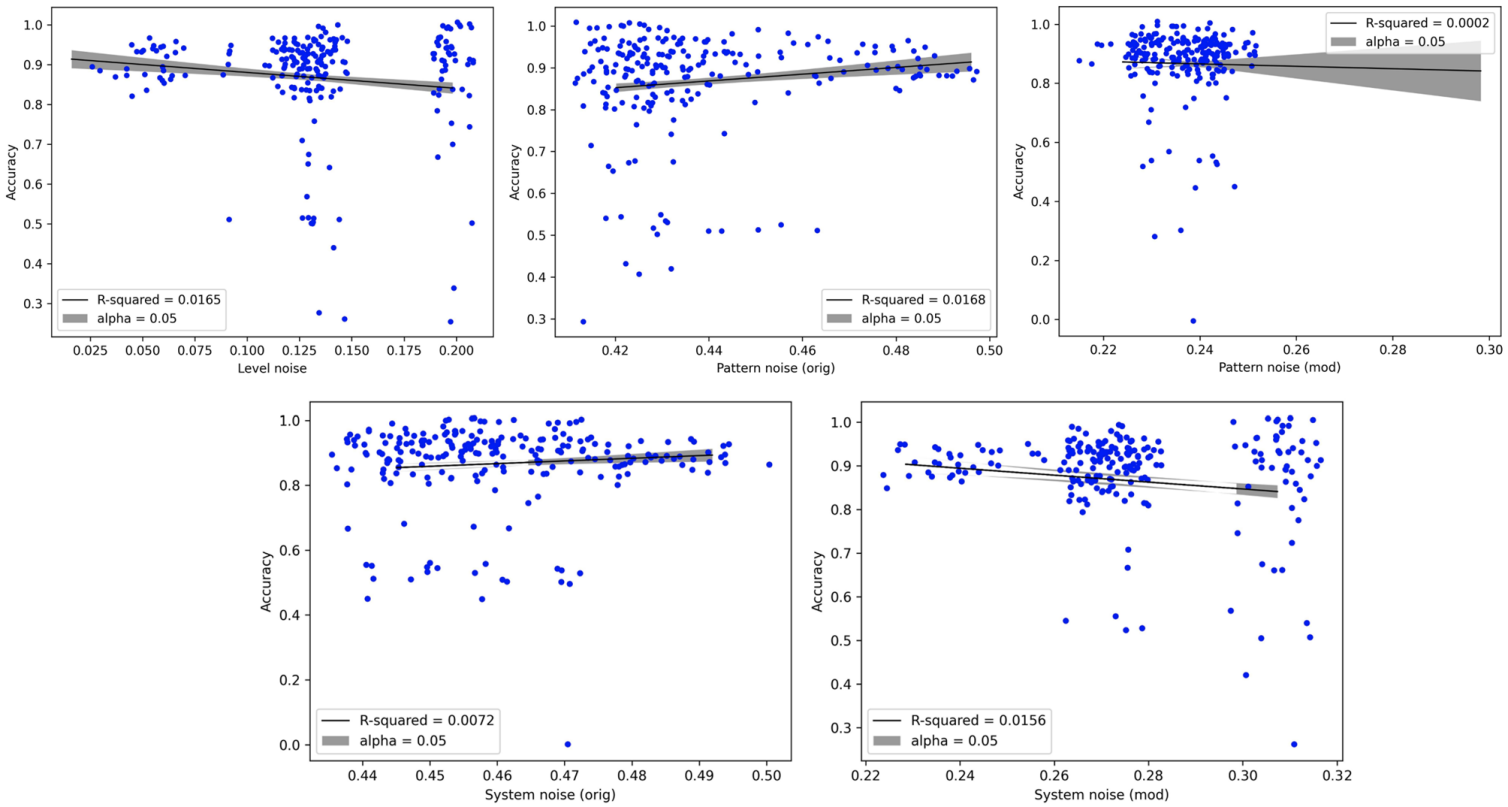
Regressions of turkers’ annotation accuracy (ComVE dataset) against the different types of noise. The points in the scatter plot are based on the same data as were used for constructing Fig. 4. *alpha* is the maximum allowable type-I error rate, which is used for plotting the 95% confidence interval (grey envelope) around the regression. Formal statistics of the regression are provided in the *Supplementary Material*. Based on the F-statistic of the regression, all regressions are significant at the 95% confidence level, despite the low goodness-of-fit, except for the *Pattern noise (mod)* regression.

We plot a similar accuracy figure for ComVE in Fig. 4. As a robustness check, we use two definitions of the ground-truth (putative, as well as modal, with the latter computed using a similar methodology as described earlier for TG-CSR). The results are more varied than those observed for TG-CSR; however, we still find a substantial confidence interval, usually spanning about 15 percentage points on the accuracy scale, in accuracy estimates of most turkers. Turkers who provide more labels (the red and orange bars on the right side of the two sub-plots) do have narrower confidence intervals, which is likely due to their providing more data. Nevertheless, even when hundreds of labels are provided, an interval of 5–10 percent remains. Otherwise, with few exceptions, most annotators are roughly consistent in their mean accuracy levels, which is above 90 percent as we would expect in a commonsense reasoning task. Furthermore, more labels alone can’t really explain the wide confidence intervals; some annotators who provided between 60–99 labels (the green bars) have narrow confidence intervals, and some annotators who provided over a hundred labels (grey bars) have wide confidence intervals. Such patterned differences are responsible for the higher pattern noise that we observed earlier in the results for RQ1.

To provide more insight into Fig. 4, the annotation accuracy results for ComVE are presented in a different way (using boxplot distributions) in Fig. 5, while controlling again for the minimum allowable labels per turker. Unlike the previous figure, note that all annotators are included in this figure, including those who provided fewer than 60 labels. We again find a qualitative similarity between the results described earlier for RQ2 and the boxplots in Fig. 5. Namely, both the mean and median accuracy is generally high (usually above 90%), but there is a significant range around the estimate, even when outliers are discarded. The range narrows as more filtering is applied, but even when the filtering threshold is 100, a range of 5% around the mean accuracy estimate can still be observed.

One question that might arise in the context of these results is: what is the specific relationship between a turker’s accuracy, and the different types of noise (level, modified pattern, and so on)? Namely, is increase in noise associated with a consistent decrease in accuracy? To investigate this relationship, which is a variant of RQ2, we can use the same results as in Fig. 2, but instead of plotting the minimum or maximum allowable labels per turker, we produce a scatter plot of their noise levels and fit a linear regression of accuracy against each type of noise to test for a trend. The results of this investigation are shown in Fig. 6. While the goodness of fit is small, it is statistically significant (as evidenced by the full statistics, provided in the supplementary material), except for the lone case of $PN^{mod}$. As expected, the trend is negative for level noise, $PN^{mod}$ (although it is not significant) and $SN^{mod}$. We also see the utility of using the modified versions of system noise: neither $SN^{orig}$ nor $PN^{orig}$ show a negative trend, which can be explained by our earlier observation that the measures of variability captured by these random variables depend on the benchmark and are not anchored (in the ideal situation of no noise being present) at 0.

Finally, considering the performance estimates of ChatGPT on the three 100-sentences sets (*no noise, moderate noise* and *noise*) sampled from ComVE, we found the accuracy to be 95%, 90% and 70% when using the modal ground-truth (mode of the five labels provided by the turkers), and 95%, 86% and 76% when using the putative ground-truth that was released with the benchmark itself. Using the unpaired z-test, we confirmed that the performance of ChatGPT on the *noise* partition was significantly different from performance on the other two partitions at a 90% confidence level, regardless of which ground-truth is used.

## Discussion

Benchmark datasets are regularly used to both train and test machine commonsense reasoning systems, a good overview of which is provided by Davis^39^. He describes different methods that can be used to develop benchmark datasets and highlights some obstacles, including issues with data quality (incorrect or inconsistent values) and the reliance on ‘single’ gold standards (derived from humans who can be very inconsistent with each other) as a sole basis for performance measurement. He also notes that for some tasks (e.g., image caption generation), comparing a machine’s performance to that of humans may be irrelevant. For tasks such as commonsense reasoning, however, a comparison to human performance (at least, on average) is crucial as it is implied that the machine is being trained to do *human* commonsense reasoning (e.g., to reason about everyday human social interaction).

In this article, we investigated two research questions that examine different aspects of noise, as defined in the behavioral psychology literature, to provide a quantitative basis for the unreliability of single ground truths in both laboratory and crowdsourced settings. Using the novel framework proposed by Kahneman, Sibony, and Sunstein^13^, we conducted a noise audit on two machine commonsense reasoning datasets: the Theoretically-Grounded Commonsense Reasoning (TG-CSR) benchmark recently developed in a laboratory setting using two different formats (multiple-choice and true–false)^16^, and the Commonsense Validation and Explanation (ComVE) dataset^29^, which is one of the earliest datasets developed specifically to evaluate commonsense reasoning (predating the large generative language models like ChatGPT).

For both benchmarks, the noise audit revealed non-trivial levels of level, pattern, and system noise values, with pattern noise tending to dominate the system noise, regardless of whether we use the original definition, or a ‘normalized’ version for which the ideal level is zero. In contrast, level noise was found to be more modest, but still non-zero. The pattern of results indicates that most observers show a similar average rating across prompts (low level noise). However, for a given prompt, different observers can vary much more significantly in their ratings (high pattern noise).

Level noise was largely consistent across both the ComVE and TG-CSR benchmarks despite the different ways in which these benchmarks were constructed and labeled. On TG-CSR, we found that the choice of the rating scale does make a difference: on three of the four contexts, level noise was significantly reduced when we switched from a four-point format to the true/false (binary) format. Binarization alone does not explain the reduction in noise, since the four-point format was binarized before analysis, ensuring that noise is measured on the same unit-scale as the true/false format. Additionally, our analysis revealed that increased level noise corresponded to a decrease in turkers’ annotation accuracy on the ComVE dataset, suggesting that small increases in level noise are still meaningful, even if the absolute numbers are lower.

Also, while independent labels acquired from humans on commonsense prompts have relatively high agreement, in realistic datasets, some prompts lead to greater individual differences (manifesting in higher level noise). For example, one of the sentences in ComVE (that a crowdsourced worker is asked to label as plausible or implausible) was ‘a dog plays volleyball’. While some people might think this is implausible, others (e.g., who have dogs and are athletic) rated it as plausible. This type of individual difference is difficult to predict in advance, but as with any real-world psychological trait or phenomenon, cannot (and should not) be eliminated from, or ignored, in the benchmark itself. Unfortunately, because of the assumption of a single ground-truth, one or the other label would ultimately be used, and subject to underlying noise. An NLP system, such as a large language model, would then suffer (or gain) in performance if it happened to disagree with the (now ‘correct’) label that was originally controversial.

Analysis using filtering on the ComVE benchmark showed that level noise can decrease if we filter the prompts, such that we only retain those prompts labeled by turkers who have provided a minimum number of labels. However, this decrease in noise is not without cost: the application of the filter leads to the elimination of labels, and consequently, prompts. In other words, we are left with a less noisy dataset, but one that is smaller (and perhaps less challenging and ‘interesting’) than the original dataset.

Our results for the second research question showed how such noise can affect accuracy even when a human is treated as a proxy for a commonsense reasoning ‘system’. The standard errors shown in Fig. 3 suggest that such accuracy estimates can vary by 5–10% in many settings, which can often make the difference between a ‘state-of-the-art’ system outperforming the previous best result. In fact, shortly after the early transformer-based language models, such as BERT^40^ and RoBERTa^41^, were released, it was not uncommon to find very narrow differences between many systems holding the top positions on a commonsense reasoning leaderboard. To take just one example, the difference between several earlier transformer-based models, such as elBERTo^42^, DeBERTa^43^, and ALBERT^44^, is less than one percent on the CosmosQA leaderboard^45^, even at the time of writing, and these models are currently all in the top twenty in the leaderboard. Evaluating ChatGPT on ComVE against two reasonable and independent ground-truths, we found a much higher difference between ChatGPT’s performance estimates, when evaluated on noisy and less noisy sentences. Because a proper noise audit has not been conducted or published for any of the benchmarks on these commonsense reasoning leaderboards, it is not possible to tell whether the difference between the leaderboard systems is occurring due to noise, or due to one system genuinely being better than another (i.e., when performance estimates are statistically adjusted for noise).

## Limitations and implications

One limitation of our results that we hope to address in future research is a more direct comparison between TG-CSR and ComVE in the online setting. This would require setting up the questions in TG-CSR as micro-tasks on a platform like Amazon Mechanical Turk, and replicating the ComVE settings as much as possible. For example, we could use the true/false modality to mirror ComVE’s plausible/implausible format, and aim to obtain five unique labels per prompt, just as with ComVE. One issue that we would need to treat with caution is that TG-CSR is divided into broad thematic areas, with many questions per area. In other words, questions in TG-CSR are not completely independent but are loosely correlated within the theme. While the questions are still meant to be answered independently, the theme may have to be modeled as a fixed effect in the online setting, especially since no one annotator will answer all questions within the thematic area because of the nature of the platform (also, different themes, perhaps owing to content-specific features like difficulty and cultural dependency, may attract variable engagement from the pool of annotators). If the noise results for ComVE can be replicated with high fidelity for TG-CSR in such an online setting, it would further lend credence to our core finding that noise is present in such benchmark datasets, including when using crowdsourced labels, and needs to be estimated and accounted for in experimental studies on commonsense reasoning that make use of them.

Along similar lines, robustness checks can also be conducted by considering alternative frameworks, besides the one used here for measuring what Kahneman et al.^13^ have termed as noise. Classical measures of inter-rater agreement, like Cohen’s kappa, could be computed using the raw data that we have provided in the supplementary files to determine consistency with pattern noise, to which it is most closely related. Similarly, a mixed linear model with explicit effects for annotators and prompts could be fit to the same data that was used in this article for measuring noise. The coefficients and residual in the regression (as well as goodness of fit) could be compared to some of our earlier results, and may provide similar insights as were reported in (for example) Fig. 6. We believe that a comparative analysis of these alternative methodologies, each seeking to measure the same underlying phenomenon of noise (at least, in principal), would provide more complete insight into the statistical properties and distribution of the phenomenon.

When considering these results, we note that statistical significance comparisons alone will not help resolve the problem of determining whether one system is ‘better’ than another system on a task (e.g., the TG-CSR vacationing abroad TF dataset). If a system A is statistically better than a system B on one ground-truth, it does not preempt A from being worse (or statistically indistinguishable) than B on another ground-truth obtained either by using a different set of annotators, or by combining the labels obtained from the same set of annotators in a different way (e.g., by using average instead of mode, by removing sentences where annotators disagree, and so on). Either way, one would expect the statistical relationship between two systems to become weaker due to this phenomenon. The real issue here is the a priori assumption of a single, fixed ground-truth, which post-hoc statistical testing cannot control for. We note that such limitations of statistical testing are well-understood in other contexts such as bias and confounding^46^ (but not, to our knowledge, in the context of hidden ground-truth noise), which also cannot be detected by ordinary statistical significance tests, such as the Student’s t-test^47,48^.

The discussion above is not meant to suggest that we get rid of benchmarking altogether, or that results from benchmarking are not valuable. However, similar to bias, noise is a problem that we ignore at our own peril, especially when we rely on narrow margins of improvement between rival ‘state-of-the-art’ AI systems to draw conclusions about progress in the field. Leaderboard results for many commonsense reasoning benchmarks, such as those hosted by the Allen Institute, have narrowed considerably, especially with the advent of language models and powerful techniques for prompting and fine-tuning. In light of the results in this paper, one pragmatic course of action, when conducting such evaluations, might be to estimate noise levels as parameters. For example, methods grounded in inferential and Bayesian statistics can be devised to estimate a noise value (and confidence intervals around that value based on the sample size), and use the value to qualify the true significance of any improvements. Additionally, researchers proposing and publishing new benchmarks could make an attempt at estimating noise levels in their ‘ground-truths,’ so that other researchers who use those benchmarks can accommodate the estimated noise levels in their analysis.

Estimated noise levels, whether obtained through sophisticated inference procedures or through an empirical study like the one conducted herein, have practical utility. For example, if level noise is estimated to be excessive (by some reasonable definition of excess), the problem could be traced to a small set of annotators, or to the annotation guidelines. It may suggest hidden faults in the labeling procedure itself (e.g., guidelines may have been written ambiguously or otherwise subject to misinterpretation). Similarly, pattern noise may be used to detect *ambiguous* prompts. Even if overall levels of pattern noise are low, we may still want to isolate or ‘re-weight’ prompts where annotators disagree so that we can evaluate the accuracy of a predictive system both with, and without, such ambiguous instances. This would allow us to determine the system’s performance more robustly than a single overall accuracy would. Furthermore, by knowing the prevalence of such ambiguous prompts, we could be assured that their presence is not the *main* reason that we are observing an improvement of one AI system over another.

Finally, we note that the study adds to a growing body of literature on the ‘fallibility’ of human judgment, and (more indirectly) to literature commenting on the limits of computational models. To cite a specific example, Salganik et al.^49^ conducted a “scientific mass collaboration” experiment, where models were built by independent teams of scientists to predict certain life outcome measures, given a training sample, on a dataset that had yielded over 750 journal publications. They found that accuracy on most of the outcome measures, even when using complex machine learning models, was relatively poor on the withheld dataset and comparable to a simple benchmark model based on regression. While there are important conceptual differences between their study, and what we are proposing here, the larger point still holds that we must be cautious in interpreting performance differences between two systems, even though one might seem more complex and ‘state-of-the-art’ than the other. Other studies in the machine learning community (particularly, computer vision) have alluded to phenomena like *dataset bias*^17,50^, which suggest that performance improvements by successively published systems on the *same* benchmark are not as significant as they appear. Our work adds to this body of knowledge by showing that noise can also make a difference, even when the task (like commonsense reasoning) seems to be simple and uncontroversial enough to not require specialized knowledge or skills on the part of annotators.

## Conclusion

In this article, we proposed and conducted a comprehensive noise audit of two benchmarks used for evaluating machine commonsense reasoning, in two different settings. Considering the results across all three research questions holistically, the experiments show that even an ‘intuitive’ human task like commonsense reasoning is not immune from noise, and that attempts to reduce noise (through processes like annotator filtering) succeed by removing potentially controversial prompts that may be more susceptible to individual (or other) differences that manifest in higher noise levels. This finding is aligned with that of Kahneman and his co-authors in their book on noise; namely, that noise may be inevitable in most reasonable real-world judgment tasks where human judgment is elicited. Not only is this true for domain-specific judgment problems (e.g., deciding what insurance premium to charge a customer) but it also applies to everyday commonsense prompts where one would theoretically (or at least, *a priori*) expect near-perfect agreement among independent human beings.

When evaluating one or more AI systems, it is essential therefore to both identify and then *adjust* the systems’ performance estimates for this noise, to the extent feasible. At minimum, our results suggest that the assumption that there is a ‘single’ ground-truth and that all prompts are equal when it comes to the level of expected noise, should be revised, or at least acknowledged, when doing comparative analyses. The claim here is *not* that ground-truths should be entirely eliminated, but rather, that benchmark designers should consider the pragmatic step of releasing, not just the one ground-truth, but the full set of labels that could then be used to conduct robustness studies (e.g., by constructing multiple ground-truths through aggregating the labels in different, but still reasonable, ways) along the lines suggested by the second research question. Furthermore, when comparing different AI systems, it may be instructive to entertain the possibility that (in part) the performance differences between rival baseline systems, may be due to label differences arising due to noise, rather than one system ‘outperforming’ another. More generally, even though our methodology was applied specifically to the evaluation of machine commonsense reasoning, it can also be used in other application areas of machine learning that rely heavily on human labels.

### Supplementary Information

Supplementary Information.

## Data Availability

All data generated or analysed during this study are included in this published article [and its supplementary information files].
