# Supplementary material for: A noise audit of human-labeled benchmarks for machine commonsense reasoning
Source: Sci Rep. 2024 Apr 14;14:8609. doi: 10.1038/s41598-024-58937-4 (PMC11016068; doi:10.1038/s41598-024-58937-4)
Supplement: Supplementary file 1 — Supplementary Information. [file 41598_2024_58937_MOESM1_ESM.pdf]

Supplementary Material

We report here the statistics of the five (*Accuracy versus Noise*) regressions in Figure 6 of the paper. Except for pattern noise (mod), all regressions are significant based on the F-statistic or (equivalently) a Student's t-test statistic on the regression coefficient.

Level Noise OLS Regression Results						
**Dep. Variable:**	y	**R-squared:**	0.016			
**Model:**	OLS	**Adj. R-squared:**	0.016			
**Method:**	Least Squares	**F-statistic:**	18.65			
**Log-Likelihood:**	666.7	**Prob (F-statistic):**	1.71E-05			
**No. Observations:**	1114	**AIC:**	-1329			
**Df Residuals:**	1112	**BIC:**	-1319			
**Df Model:**	1					
**Covariance Type:**	nonrobust					
	**coef**	**std err**	**t**	**P>|t|**	**[0.025**	**0.975]**
**const**	0.9203	0.013	71.011	0	0.895	0.946
**x**	-0.398	0.092	-4.318	0	-0.579	-0.217
**Omnibus:**	636.268	**Durbin-Watson:**	1.881			
**Prob(Omnibus):**	0	**Jarque-Bera (JB):**	4829.844			
**Skew:**	-2.6	**Prob(JB):**	0			
**Kurtosis:**	11.776	**Cond. No.**	23.5			

Pattern Noise (orig) OLS Regression Results						
**Dep. Variable:**	y	**R-squared:**	0.017			
**Model:**	OLS	**Adj. R-squared:**	0.016			
**Method:**	Least Squares	**F-statistic:**	19.04			
**Log-Likelihood:**	669.07	**Prob (F-statistic):**	1.40E-05			
**No. Observations:**	1116	**AIC:**	-1334			
**Df Residuals:**	1114	**BIC:**	-1324			
**Df Model:**	1					

**Covariance Type:**	nonrobust					
	**coef**	**std err**	**t**	**P>|t|**	**[0.025**	**0.975]**
**const**	0.5136	0.081	6.332	0	0.354	0.673
**x**	0.8069	0.185	4.364	0	0.444	1.17
**Omnibus:**	648.995	**Durbin-Watson:**	1.88			
**Prob(Omnibus):**	0	**Jarque-Bera (JB):**	5148.267			
**Skew:**	-2.642	**Prob(JB):**	0			
**Kurtosis:**	12.099	**Cond. No.**	55.4			

**Pattern Noise (mod) OLS Regression Results**						
**Dep. Variable:**	y	**R-squared:**	0			
**Model:**	OLS	**Adj. R-squared:**	-0.001			
**Method:**	Least Squares	**F-statistic:**	0.2354			
**Log-Likelihood:**	657.55	**Prob (F-statistic):**	0.628			
**No. Observations:**	1114	**AIC:**	-1311			
**Df Residuals:**	1112	**BIC:**	-1301			
**Df Model:**	1					
**Covariance Type:**	nonrobust					
	**coef**	**std err**	**t**	**P>|t|**	**[0.025**	**0.975]**
**const**	0.9637	0.199	4.838	0	0.573	1.355
**x**	-0.4086	0.842	-0.485	0.628	-2.061	1.244
**Omnibus:**	675.496	**Durbin-Watson:**	1.849			
**Prob(Omnibus):**	0	**Jarque-Bera (JB):**	5671.948			
**Skew:**	-2.769	**Prob(JB):**	0			
**Kurtosis:**	12.567	**Cond. No.**	221			

**System Noise (orig) OLS Regression Results**						
**Dep. Variable:**	y	**R-squared:**	0.007			

**Model:**	OLS	**Adj. R-squared:**	0.006			
**Method:**	Least Squares	**F-statistic:**	8.067			
**Log-Likelihood:**	661.46	**Prob (F-statistic):**	4.59E-03			
**No. Observations:**	1114	**AIC:**	-1319			
**Df Residuals:**	1112	**BIC:**	-1309			
**Df Model:**	1					
**Covariance Type:**	nonrobust					
	**coef**	**std err**	**t**	**P>|t|**	**[0.025**	**0.975]**
**const**	0.4876	0.134	3.647	0	0.225	0.75
**x**	0.8245	0.29	2.84	0.005	0.255	1.394
**Omnibus:**	673.426	**Durbin-Watson:**	1.861			
**Prob(Omnibus):**	0	**Jarque-Bera (JB):**	5728.524			
**Skew:**	-2.751	**Prob(JB):**	0			
**Kurtosis:**	12.651	**Cond. No.**	87.8			

**System Noise (mod) OLS Regression Results**						
**Dep. Variable:**	y	**R-squared:**	0.016			
**Model:**	OLS	**Adj. R-squared:**	0.015			
**Method:**	Least Squares	**F-statistic:**	17.63			
**Log-Likelihood:**	666.2	**Prob (F-statistic):**	2.90E-05			
**No. Observations:**	1114	**AIC:**	-1328			
**Df Residuals:**	1112	**BIC:**	-1318			
**Df Model:**	1					
**Covariance Type:**	nonrobust					
	**coef**	**std err**	**t**	**P>|t|**	**[0.025**	**0.975]**
**const**	1.0846	0.052	20.872	0	0.983	1.187
**x**	-0.793	0.189	-4.199	0	-1.164	-0.422
**Omnibus:**	637.161	**Durbin-Watson:**	1.88			
**Prob(Omnibus):**	0	**Jarque-Bera (JB):**	4836.078			
**Skew:**	-2.605	**Prob(JB):**	0			

**Kurtosis:**	11.778	**Cond. No.**	50.9			
